# Treatment of Stage III Grade C Periodontitis With Smoking Cessation Care and Periodontal Surgery Including Regenerative Therapy: A Case Report With a Two-Year Follow-Up

**DOI:** 10.7759/cureus.85449

**Published:** 2025-06-06

**Authors:** Rio Hisanaga-Mokudai, Kouki Yoshikawa, Atsushi Saito

**Affiliations:** 1 Department of Periodontology, Tokyo Dental College, Tokyo, JPN

**Keywords:** guided tissue regeneration, open flap debridement, periodontal regenerative therapy, rhfgf-2, smoking cessation

## Abstract

We report a case of generalized chronic periodontitis requiring periodontal treatment, including smoking cessation care and surgical procedures. With a chief complaint of swelling and pain in the gingiva around #47, a 41-year-old man visited the Tokyo Dental College Suidobashi Hospital. The patient had been smoking cigarettes for 31 years. The initial periodontal examination identified a probing depth (PD) of ≥4 mm in 72.6% of sites and bleeding on probing (BOP) in 45.2% of sites. Radiographic examination demonstrated bone resorption extending to the root apex in #37 and 47, angular bone resorption in #17 and 24, furcation radiolucency in #17, 27, 37, and 47, and horizontal resorption in other areas. Following the clinical diagnosis of stage III grade C periodontitis, non-surgical therapy consisting of smoking cessation care, plaque control, scaling and root planing (SRP), and placement of occlusal splint was performed. Teeth #37 and 47 were extracted due to bone resorption extending to the root apex. The patient had achieved smoking cessation by the end of the non-surgical therapy. Subsequently, surgical periodontal treatment was performed at the selected site. Periodontal regenerative therapy using recombinant human fibroblast growth factor (rhFGF)-2 was carried out for #14 and 24. Guided tissue regeneration (GTR) was implemented for #17. Open flap debridement was performed in #16, 23, 25, 26, and 27 to facilitate the reduction of periodontal pockets. Following periodontal re-evaluation, supportive periodontal therapy (SPT) was initiated. The patient maintained smoking cessation after the non-surgical therapy, and periodontal stability was observed over a two-year period. Periodontal therapy contributed to a marked improvement in the patient’s oral health-related quality of life.

## Introduction

Cigarette smoking is among the most significant risk factors for the development and progression of periodontitis. From both epidemiological and biological aspects, there is ample evidence documenting the adverse effects of cigarette smoking on periodontal status. For instance, in a study of 240 dental patients, smokers had 2.7 times and former smokers 2.3 times greater probabilities of having established periodontal disease than non-smokers, independent of age, sex, and plaque index [1]. In addition, smoking exerts direct effects on the human microbiome, as well as indirect effects mediated by mechanisms such as immunosuppression, oxygen deprivation, and biofilm formation [2]. In addition, smoking exerts direct and indirect effects on the human microbiome [3].

However, the risk for periodontitis incidence and progression could be reversed after smoking cessation to a similar level as that of never smokers [4]. A recent study further demonstrated the effectiveness of smoking cessation interventions, highlighting the importance of behavioral support within periodontal therapy [5].

Here, we report a case of chronic periodontitis in a patient with a history of smoking, treated with periodontal therapy that included smoking cessation care and periodontal surgery. The periodontal condition has been successfully maintained for over two years with continued smoking cessation.

## Case presentation

First visit examination

A 41-year-old man presented to the Clinic of Conservative Dentistry, Tokyo Dental College Suidobashi Hospital, in October 2019, complaining of swelling and pain of the gingiva around #47. The patient was in good general health. He had been smoking since he was 10 years old (approximately 20 cigarettes/day). Although he visited a smoking cessation clinic due to rising cigarette prices, he was unable to quit smoking.

**Figure 1:**
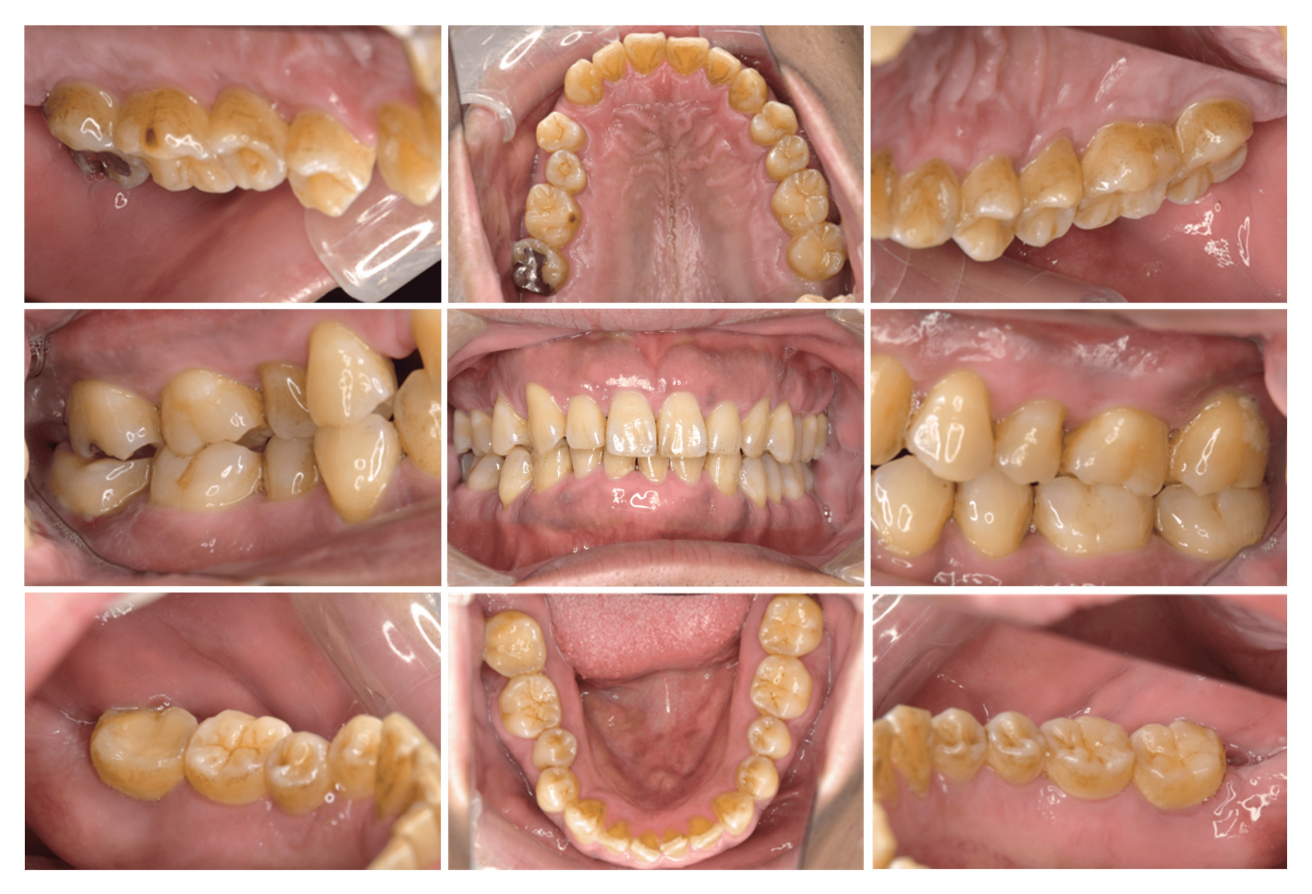
Oral view at the first visit

Although he received dental caries treatment of #17 at a local dental office when he was 30 years old, he had no experience with periodontal treatment. In September 2019, the patient noticed spontaneous pain in #47 and received pulpectomy treatment of #47 at a local dental office. However, the swelling and pain of the gingiva around #47 did not improve; thus, he visited our clinic. An intraoral view taken at the initial visit is presented in Figure 1.

Initial examination revealed inflammation of the gingiva around #47. Premature occlusal contact and balancing contact were observed in #27 and 37. The patient was aware of sleep bruxism and daytime clenching.

**Figure 2:**
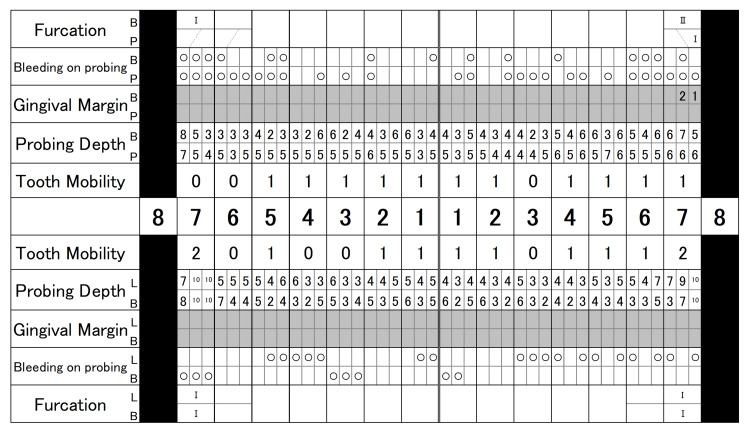
Periodontal examination at the first visit

Periodontal examination (Figure 2) revealed that 48.8% of sites had a probing depth (PD) of 4-5 mm and 23.8% a PD of ≥6 mm.

**Figure 3:**
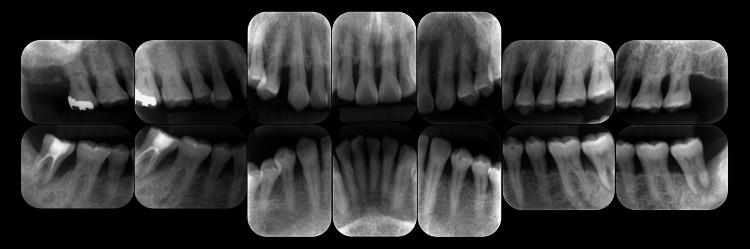
Radiographic view at the first visit

Bleeding on probing (BOP) was detected at 45.2% of the examined sites, and the periodontal inflamed surface area (PISA) was calculated to be 1807.4 mm². Tooth mobility was observed in #11-15, 21, 22, 24-27, 31, 32, 34-37, 41, 42, 45, and 47. The O’Leary plaque control record (PCR) indicated a plaque control level of 68.8%. As shown in Figure 3, radiographic examination demonstrated alveolar bone resorption that had extended to the root apex in #37 and 47, angular bone defects in #17, 24, 37, and 47, and furcation radiolucency in #17, 27, 37, and 47.

According to Lindhe and Nyman’s classification [6], the furcation involvement was diagnosed as degree I for #17, 37, and 47, and degree II for #27. To evaluate patient-reported outcomes, oral health-related quality of life (QoL) was assessed using the Japanese version of Oral Health-Related Quality of Life (OHRQL-J) [7], with a total score of 19.

Diagnosis

The clinical diagnosis was severe generalized chronic periodontitis, stage III, grade C [8].

Treatment plan

The proposed treatment plan was explained to the patient, and informed consent was obtained. The patient did not wish for comprehensive orthodontic treatment, placement of a removable partial denture, or placement of a dental implant. Hence, the risk of extrusion of #17 and 27 was also explained to the patient.

The treatment plan was as follows. The first course of action was initial periodontal therapy, which comprised extractions of #37 and 47, smoking cessation care, oral hygiene instruction, scaling and root planing (SRP), and placement of an occlusal splint. This was followed by re-evaluation. Then, periodontal surgery was planned for sites with a PD of ≥4 mm, contingent on the patient’s successful smoking cessation. Sites with a PD of ≥6 mm and/or bone defects were predicted to retain deep pockets after initial periodontal therapy. Open flap debridement was planned for the sites with a PD of ≥4 mm, and periodontal regenerative therapy was planned for #17 and 24. This was again followed by re-evaluation and then supportive periodontal therapy (SPT) or maintenance.

Clinical procedure and outcomes

Treatment Process

An outline of the treatment process is shown in Table 1.

**Table 1:** Treatment process SRP: scaling and root planing, GTR: guided tissue regeneration, rhFGF-2: recombinant human fibroblast growth factor-2

Period	Treatment
October 2019	Initial periodontal therapy, extraction (#37, 47), smoking cessation care, plaque control, quadrant-based SRP, occlusal splint
November 2020	Re-evaluation, surgical periodontal therapy, periodontal regenerative therapy with rhFGF-2 (#24), open flap debridement (#23, 25), periodontal regenerative therapy with rhFGF-2 (#14), GTR (#17), open flap debridement (#16), open flap debridement (#26, 27)
January 2022 to present	Re-evaluation, supportive periodontal therapy, oral hygiene instruction, professional tooth cleaning

Initial periodontal therapy: After obtaining informed consent for the proposed treatment plan, #37 and 47 were extracted due to acute symptoms and bone defects extending to the apex. The instructions were given on smoking cessation and maintaining oral hygiene. A series of quadrant-based SRP was performed. An occlusal splint was fabricated, and the patient was instructed to wear it exclusively at night. During the day, the patient was instructed to consciously avoid occlusal contact to reduce clenching. The patient stopped smoking completely in 10 months from the first visit.

Re-evaluation: Subsequent re-evaluation revealed a reduction in the PCR score to 18.3%. Sites had a PD of ≥4 mm and BOP decreased to 16% and 22.4%, respectively. PISA was 381.2 mm^2^. The total OHRQL-J score was 5. Although initial periodontal therapy improved periodontitis, we decided to perform periodontal surgery on the sites that remained a PD of ≥4 mm in this case.

**Figure 4:**
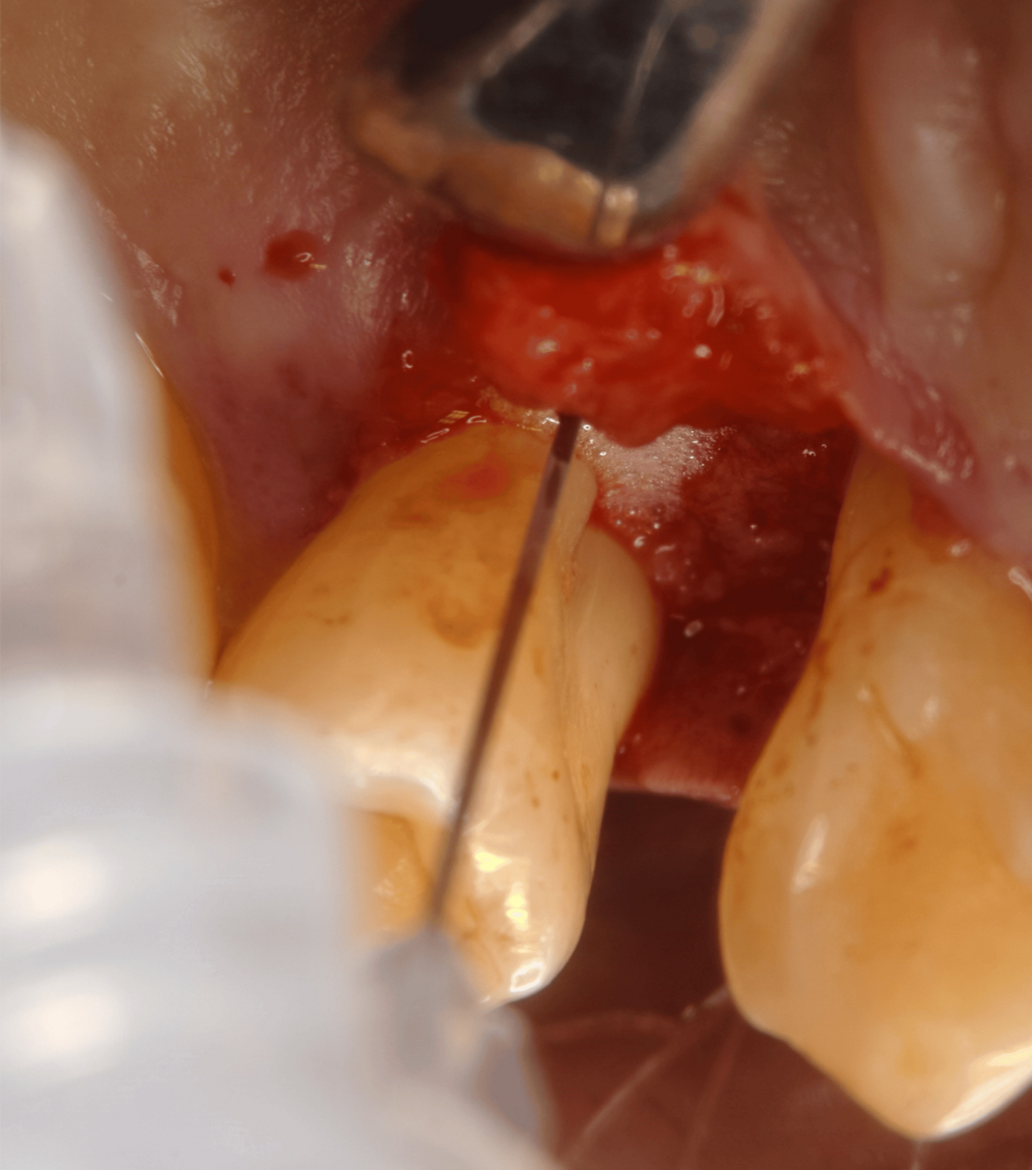
Periodontal surgery (#14 periodontal regenerative therapy with rhFGF-2) rhFGF-2: recombinant human fibroblast growth factor-2

**Figure 5:**
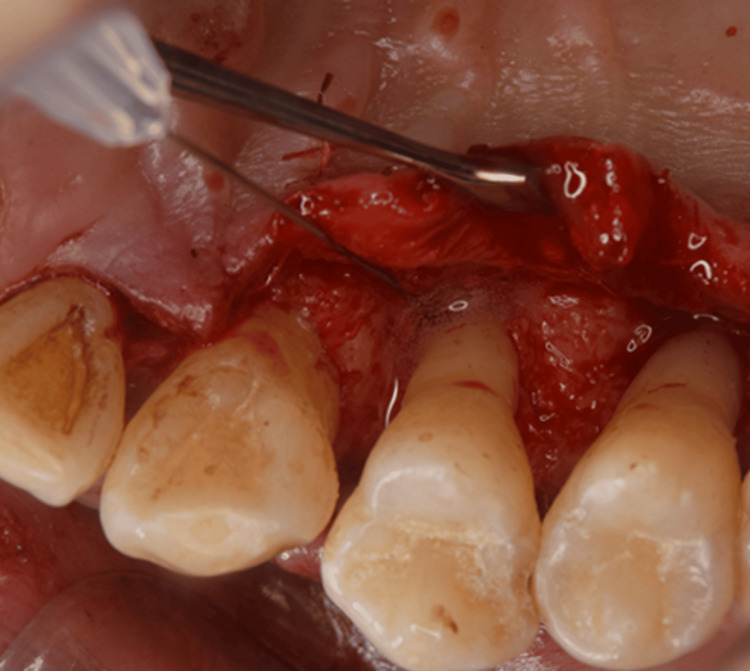
Periodontal surgery (#24 periodontal regenerative therapy with rhFGF-2, and #23 and 25 open flap debridement) rhFGF-2: recombinant human fibroblast growth factor-2

**Figure 6:**
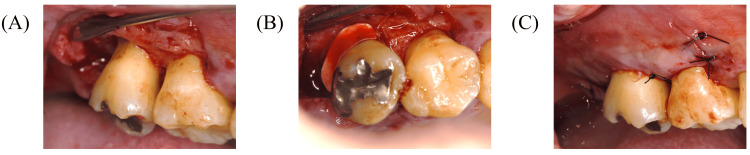
Periodontal surgery (#17 GTR and #16 open flap debridement) Periodontal surgery (#17 GTR and #16 open flap debridement): (A) after debridement, (B) placement of membrane, and (C) after suturing. GTR: guided tissue regeneration

**Figure 7:**
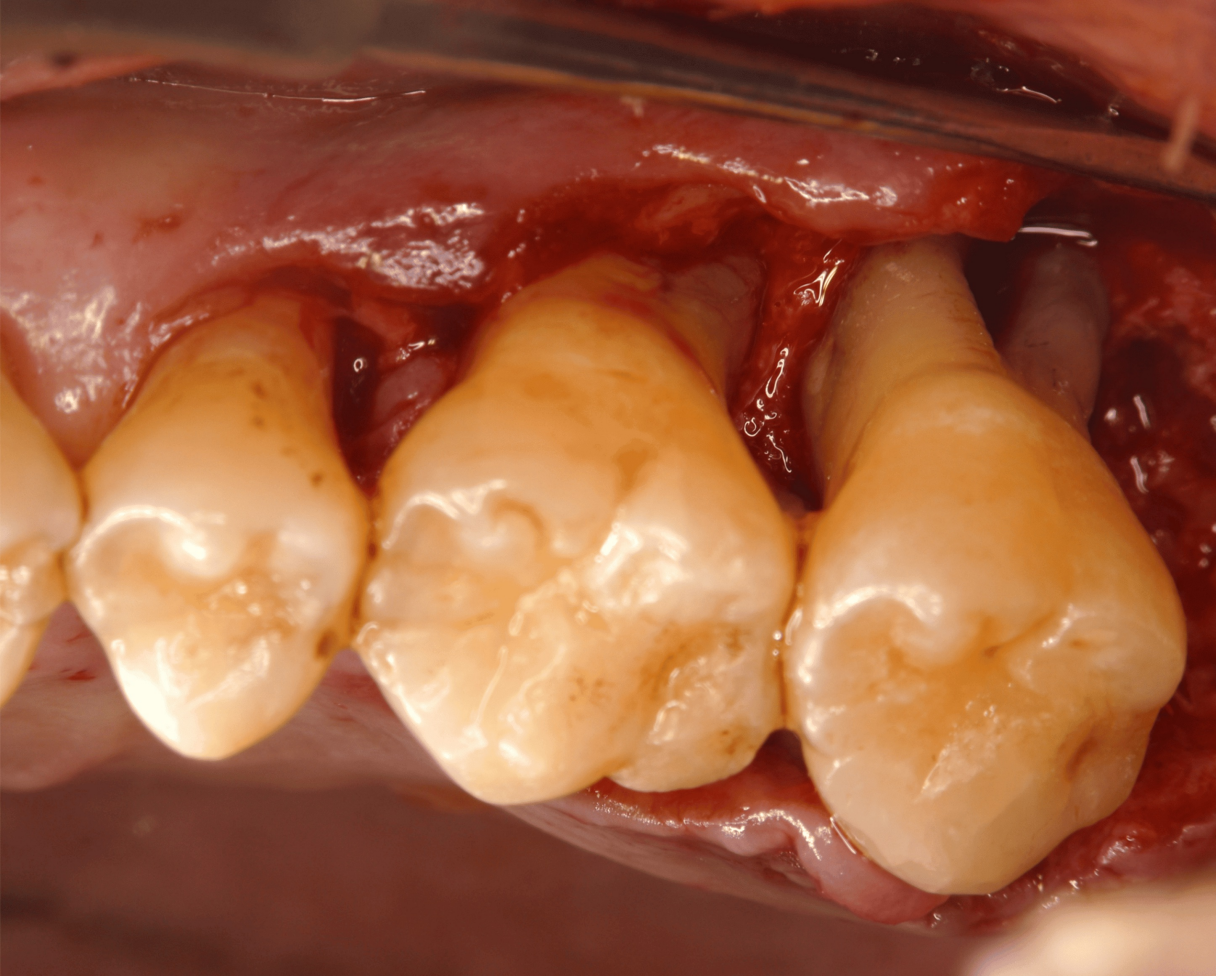
Periodontal surgery (#26 and 27 open flap debridement)

Periodontal surgery: Based on these findings, the necessity and options for periodontal surgery were explained to the patient. At three months following smoking cessation, a series of periodontal surgeries was performed (Figures 4-7). Open flap debridement was implemented for #16, 23, 25, 26, and 27 to reduce periodontal pockets. In addition, the patient received recombinant human fibroblast growth factor (rhFGF)-2 therapy for intrabony defects of #14 and 24, and guided tissue regeneration for furcation defects of #17.

Re-evaluation: An improvement was observed in gingival inflammation and PD. The periodontal conditions were deemed stable, and the patient was placed on SPT. The total OHRQL-J score was 4, indicating an improvement in QoL compared to the initial visit.

**Figure 8:**
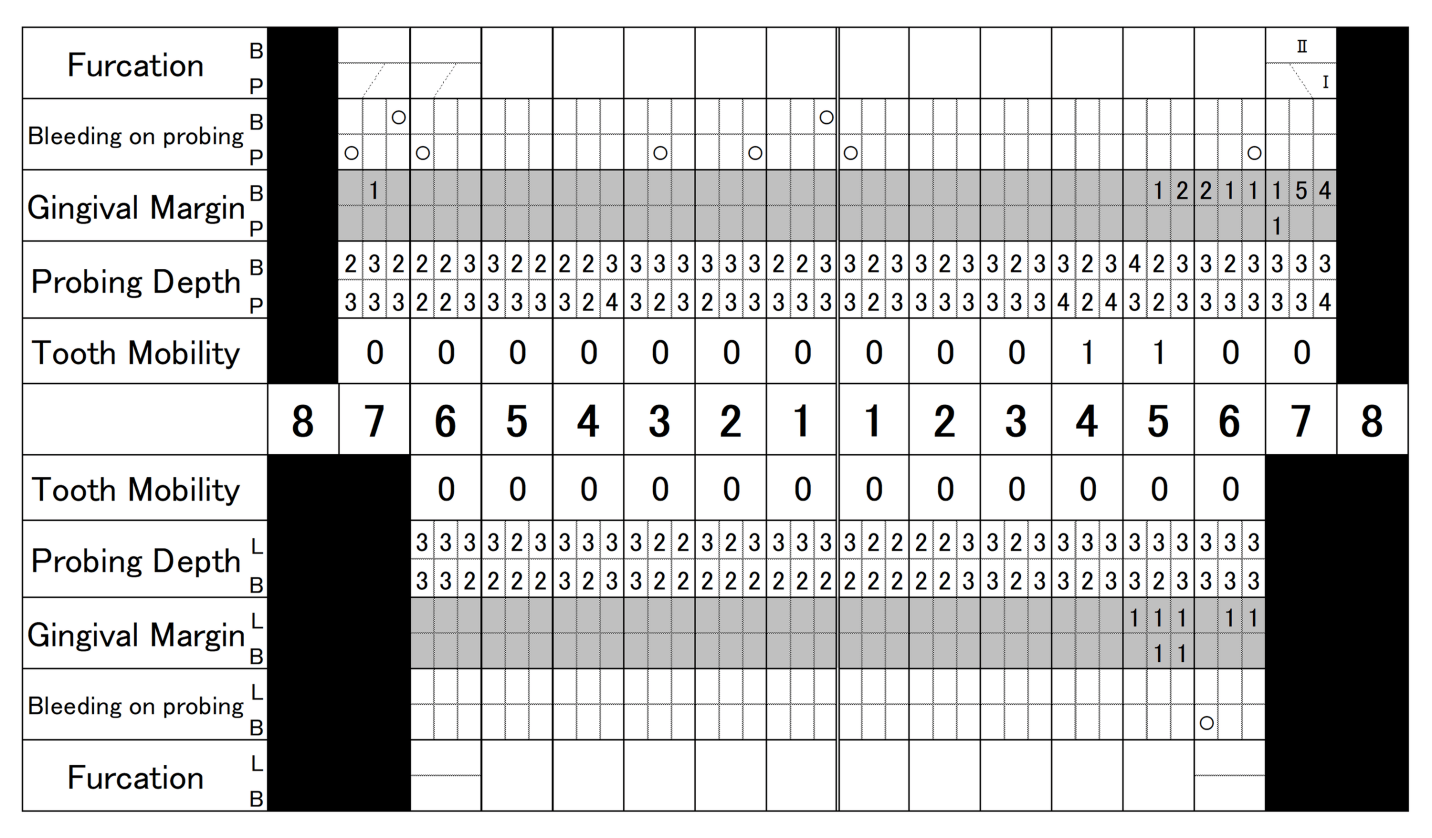
Periodontal examination at two years of SPT SPT: supportive periodontal therapy

**Figure 9:**
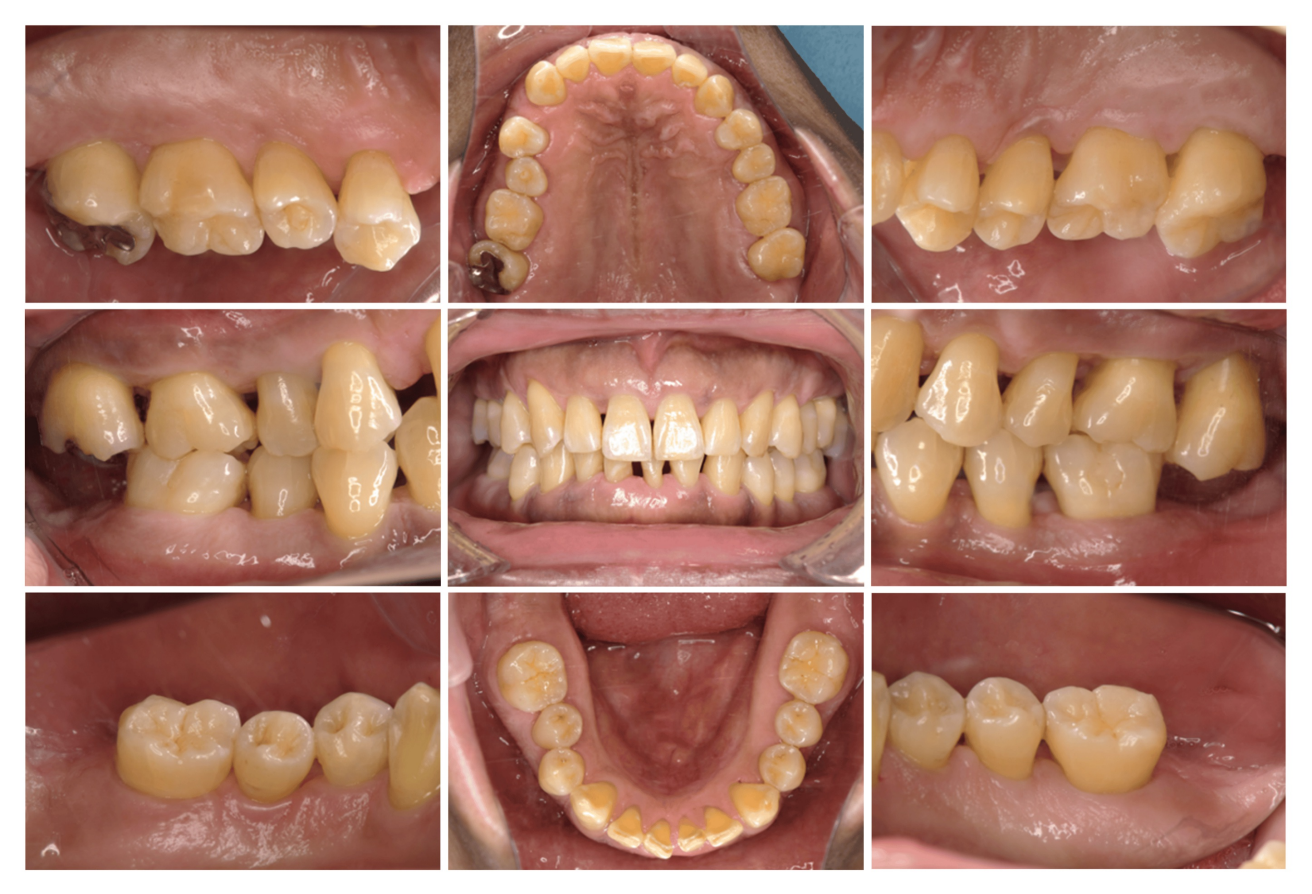
Oral view at two years of SPT SPT: supportive periodontal therapy

**Figure 10:**
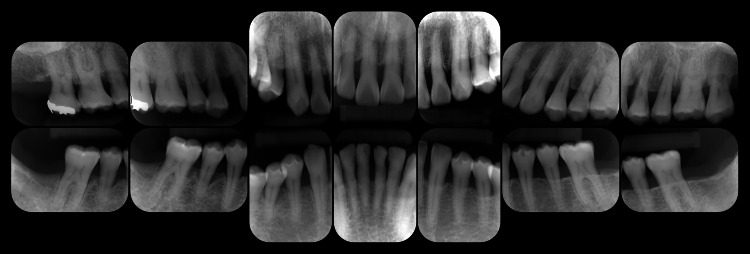
Radiographic view at two years of SPT SPT: supportive periodontal therapy

SPT: At two years from the start of SPT, periodontal conditions remained stable in most of the teeth (Figures 8-10).

Attachment gains of 2.0-3.0 mm were obtained two years after surgery (Table 2).

**Table 2:** Changes in clinical parameters PD: probing depth, CAL: clinical attachment level, MB: mesio-buccal, DB: disto-buccal, MP: mesio-palatal

Sites		Pre-op	6 months	9 months	12 months	24 months
#24 MP	PD (mm)	6	4	4	4	4
	CAL (mm)	6	4	4	4	4
#14 MB	PD (mm)	6	3	3	3	3
	CAL (mm)	6	3	3	3	3
#17 DB	PD (mm)	8	3	3	2	2
	CAL (mm)	8	3	3	2	2
#27 MB	PD (mm)	6	3	3	3	3
	CAL (mm)	6	4	4	4	4

Sites showed that a PD of ≥4 mm was 3.2%, and BOP was observed at 6.1% of sites. PISA was 78.0 mm^2^. The total OHRQL-J score was 2. Professional tooth cleaning, including subgingival plaque control, was performed on teeth with a PD of ≥4 mm at each visit for SPT. The occlusal splint was frequently checked and adjusted to minimize occlusal trauma.

## Discussion

At the initial visit, the patient presented 1807.4 mm² of PISA. Leira et al. classified 80 individuals, including smokers, into three groups based on the severity of periodontitis [9]. The mean PISA value for the group with severe periodontitis was 2309.42 ± 587.69 mm². Generally, smokers have been shown to have significantly lower gingival bleeding compared to non-smokers [10]. In this case, smoking may have masked the signs of periodontal inflammation, leading to an underestimation of the severity of periodontitis.

Periodontal surgery was initially planned only if the patient successfully quit smoking, as smoking has been shown to negatively impact clinical outcomes in surgical interventions. At the initial visit, we advised the patient to attend a smoking cessation clinic; however, he had no intention of quitting and did not attend the clinic. Therefore, based on the “5 Rs” for enhancing motivation to quit tobacco (Relevance, Risks, Rewards, Roadblocks, and Repetition) [11], we decided to provide smoking cessation support directly. For example, we explained the relationship between his current symptoms and smoking (Relevance) and discussed the risks of passive smoking to his wife (Risks). We then presented the possibility of improved breath and clothing odor with cessation (Rewards) and the financial benefits (Rewards). As his cigarette consumption began to decrease, we explained challenges faced during cessation, such as nicotine withdrawal symptoms and social pressure to smoke at work (Roadblocks). These smoking-related topics were addressed at every visit before treatment (Repetition). One month after beginning smoking cessation support, the patient started showing a willingness to quit smoking. After three months, his daily cigarette use decreased to 2-3 cigarettes, and complete smoking cessation was achieved within 10 months from the initial visit.

In this case, smoking cessation was achieved during initial periodontal therapy, and periodontal surgery was performed three months after cessation. Open flap debridement was performed for #16, 23, 25, 26, and 27 to reduce periodontal pockets. Notably, #27 presented with a degree I-II furcation involvement and horizontal bone loss. Due to the reduced bone levels on the mesial and distal aspects, a favorable outcome through periodontal tissue regeneration was not anticipated. Open flap debridement has been reported to result in an average gingival recession of 1.87 mm at 12 months postoperatively [12], raising concerns about root surface exposure in the furcation area. However, the efficacy of subgingival calculus removal in multi-rooted teeth is reported to be limited [13]. Therefore, open flap debridement was selected as the treatment modality for #27. After surgery, buccal furcation exposure and gingival recession were observed; however, hypersensitivity was not reported. Supportive periodontal therapy (SPT) is ongoing, with continued emphasis on plaque control.

The patient received rhFGF-2 therapy for intrabony defects of #14 and 24, and guided tissue regeneration for furcation defects of #17. Several studies have demonstrated the efficacy of rhFGF-2 in promoting periodontal tissue regeneration [14,15]. One earlier clinical trial confirmed the efficacy and safety of rhFGF-2 therapy, showing that it yielded greater bone fill than enamel matrix derivatives [16]. Therefore, FGF-2 was selected as the signaling molecule in the present case. In this case, an attachment gain of 2.0-3.0 mm was obtained two years after surgery. Aoki et al. reported a mean gain in CAL of 3.1 ± 1.5 mm at two years postoperatively with the application of rhFGF-2 [17]. Although the present case showed a slightly lower CAL gain, a comparable level of improvement was observed.

Additionally, in the present case, GTR was performed at the buccal and distal surface of #17. The preoperative probing depth at this site was 8 mm, and the bone defect exhibited a two- to three-walled configuration with degree I furcation involvement. A previous study showed that the mean gain in CAL at 10 years was 2.8 ± 1.2 mm following the treatment of intrabony defects with GTR [18]. A clinical attachment gain of 6.0 mm was obtained at two years postoperatively, indicating a favorable clinical outcome.

In terms of periodontal healing after surgery, sufficient blood flow is considered to be crucial. Smokers undergoing smoking cessation support, gingival bleeding, and gingival blood flow, as well as gingival crevicular fluid flow, increase and normalize toward non-smoker levels after quitting [19,20]. Morozumi et al. reported that gingival blood flow rates in former smokers were significantly higher three weeks after quitting smoking [19]. Similarly, Nair et al. reported a significant reduction in the proportion of tooth sites with plaque, but a significant increase in the proportion of tooth sites with bleeding on probing 4-6 weeks after quitting smoking [20]. Therefore, from the perspective of blood flow, it is considered desirable to perform periodontal surgery at least one month after smoking cessation. In the present case, periodontal surgeries were initiated three months after smoking cessation, and favorable periodontal healing was observed at the treated sites. However, since smoking affects not only blood flow but also cellular dynamics and the host immune response, the timing of surgical intervention following smoking cessation should be carefully considered. This case demonstrates that successful smoking cessation can contribute to favorable clinical outcomes in periodontal therapy, including surgical intervention.

Emerging techniques in splint fabrication using virtual surgical planning have shown promise in improving accuracy and efficiency in complex maxillofacial cases and may offer future value in designing customized occlusal appliances for periodontal therapy as well [21].

## Conclusions

This case illustrates the critical role of smoking cessation in enabling successful periodontal surgery that would have been discouraged in an active smoker. Following cessation, comprehensive periodontal treatment including regenerative therapy yielded favorable clinical outcomes maintained at two-year follow-up. These findings demonstrate that periodontal stability is achievable even in former long-term smokers when treatment protocols incorporate structured smoking cessation support. The case underscores the importance of addressing modifiable risk factors such as smoking as an integral component of comprehensive periodontal therapy, potentially expanding treatment options and improving long-term outcomes for patients with histories of tobacco use.

## References

[1] Calsina G, Ramón JM, Echeverría JJ (2002). Effects of smoking on periodontal tissues. J Clin Periodontol.

[2] Machtei EE, Dunford R, Hausmann E (1997). Longitudinal study of prognostic factors in established periodontitis patients. J Clin Periodontol.

[3] Buduneli N (2021). Environmental factors and periodontal microbiome. Periodontol 2000.

[4] Leite FR, Nascimento GG, Baake S, Pedersen LD, Scheutz F, López R (2019). Impact of smoking cessation on periodontitis: a systematic review and meta-analysis of prospective longitudinal observational and interventional studies. Nicotine Tob Res.

[5] Ramseier CA, Woelber JP, Kitzmann J, Detzen L, Carra MC, Bouchard P (2020). Impact of risk factor control interventions for smoking cessation and promotion of healthy lifestyles in patients with periodontitis: a systematic review. J Clin Periodontol.

[6] Nyman S, Lindhe J (1989). Examination of patients with periodontal disease. Textbook of Clinical Periodontology.

[7] Saito A, Hosaka Y, Kikuchi M (2010). Effect of initial periodontal therapy on oral health-related quality of life in patients with periodontitis in Japan. J Periodontol.

[8] Papapanou PN, Sanz M, Buduneli N (2018). Periodontitis: consensus report of workgroup 2 of the 2017 World Workshop on the Classification of Periodontal and Peri-Implant Diseases and Conditions. J Clin Periodontol.

[9] Leira Y, Martín-Lancharro P, Blanco J (2018). Periodontal inflamed surface area and periodontal case definition classification. Acta Odontol Scand.

[10] Preber H, Bergström J (1985). Occurrence of gingival bleeding in smoker and non-smoker patients. Acta Odontol Scand.

[11] Anderson JE, Jorenby DE, Scott WJ, Fiore MC (2002). Treating tobacco use and dependence: an evidence-based clinical practice guideline for tobacco cessation. Chest.

[12] Froum SJ, Weinberg MA, Tarnow D (1998). Comparison of bioactive glass synthetic bone graft particles and open debridement in the treatment of human periodontal defects. A clinical study. J Periodontol.

[13] Fleischer HC, Mellonig JT, Brayer WK, Gray JL, Barnett JD (1989). Scaling and root planing efficacy in multirooted teeth. J Periodontol.

[14] Murakami S (2011). Periodontal tissue regeneration by signaling molecule(s): what role does basic fibroblast growth factor (FGF-2) have in periodontal therapy?. Periodontol 2000.

[15] Seshima F, Bizenjima T, Aoki H (2022). Periodontal regenerative therapy using RHFGF-2 and deproteinized bovine bone mineral versus RHFGF-2 alone: 4-year extended follow-up of a randomized controlled trial. Biomolecules.

[16] Kitamura M, Akamatsu M, Kawanami M (2016). Randomized placebo-controlled and controlled non-inferiority phase III trials comparing trafermin, a recombinant human fibroblast growth factor 2, and enamel matrix derivative in periodontal regeneration in intrabony defects. J Bone Miner Res.

[17] Aoki H, Bizenjima T, Seshima F (2021). Periodontal surgery using rhFGF-2 with deproteinized bovine bone mineral or rhFGF-2 alone: 2-year follow-up of a randomized controlled trial. J Clin Periodontol.

[18] Sculean A, Kiss A, Miliauskaite A, Schwarz F, Arweiler NB, Hannig M (2008). Ten-year results following treatment of intra-bony defects with enamel matrix proteins and guided tissue regeneration. J Clin Periodontol.

[19] Morozumi T, Kubota T, Sato T, Okuda K, Yoshie H (2004). Smoking cessation increases gingival blood flow and gingival crevicular fluid. J Clin Periodontol.

[20] Nair P, Sutherland G, Palmer RM, Wilson RF, Scott DA (2003). Gingival bleeding on probing increases after quitting smoking. J Clin Periodontol.

[21] Koenig ZA, Lokant BT, Weaver S, Brooke SM, Uygur HS (2024). Surgical guide splint fabrication via virtual surgical planning for complex mandible fractures in the trauma setting. J Craniofac Surg.

